# Factors associated with blood mercury concentrations and their interactions with three glutathione S-transferase genes (*GSTT1*, *GSTM1*, and *GSTP1*): an exposure assessment study of typically developing Jamaican children

**DOI:** 10.1186/s12887-023-04452-w

**Published:** 2024-01-04

**Authors:** Sheikh Farzana Zaman, Maureen Samms-Vaughan, Sepideh Saroukhani, Jan Bressler, Manouchehr Hessabi, Megan L. Grove, Sydonnie Shakespeare Pellington, Katherine A. Loveland, Mohammad H. Rahbar

**Affiliations:** 1 Department of Epidemiology, Human Genetics, and Environmental Sciences (EHGES), School of Public Health, The University of Texas Health Science Center at Houston, Houston, TX 77030 USA; 2 Biostatistics/Epidemiology/Research Design (BERD) Component, Center for Clinical and Translational Sciences (CCTS), The University of Texas Health Science Center at Houston, Houston, TX 77030 USA; 3 Department of Child & Adolescent Health, The University of the West Indies (UWI), Mona Campus, Kingston 7, Kingston, Jamaica; 4 Department of Internal Medicine, Division of Clinical and Translational Sciences, McGovern Medical School, The University of Texas Health Science Center at Houston, Houston, TX 77030 USA; 5 Human Genetics Center, School of Public Health, The University of Texas Health Science Center at Houston, Houston, TX 77030 USA; 6 Louis A Faillace, MD, Department of Psychiatry and Behavioral Sciences, McGovern Medical School, The University of Texas Health Science Center at Houston, Houston, TX 77054 USA

**Keywords:** Blood Hg concentrations, Glutathione S-transferase (GST) genes, Fish and seafood consumption, Interaction, Effect modifier, Jamaica

## Abstract

**Background:**

Jamaican soil is abundant in heavy metals including mercury (Hg). Due to availability and ease of access, fish is a traditional dietary component in Jamaica and a significant source of Hg exposure. Mercury is a xenobiotic and known neuro-toxicant that affects children's neurodevelopment. Human glutathione S-transferase (GST) genes, including *GSTT1, GSTM1*, and *GSTP1*, affect Hg conjugation and elimination mechanisms.

**Methods:**

In this exposure assessment study we used data from 375 typically developing (TD) 2–8-year-old Jamaican children to explore the association between environmental Hg exposure, GST genes, and their interaction effects on blood Hg concentrations (BHgCs). We used multivariable general linear models (GLMs).

**Results:**

We identified the child’s age, consumption of saltwater fish, canned fish (sardine, mackerel), string beans, grain, and starches (pasta, macaroni, noodles) as the environmental factors significantly associated with BHgCs (all *P* < 0.05). A significant interaction between consumption of canned fish (sardine, mackerel) and *GSTP1* in relation to BHgC using either a co-dominant or recessive genetic model (overall interaction *P* = 0.01 and *P* < 0.01, respectively) indicated that consumption of canned fish (sardine, mackerel) was significantly associated with higher mean BHgC only among children with the *GSTP1* Ile105Val, Ile/Ile [Ratio of mean Hg (95% CI) = 1.59 (1.09, 2.32), *P* = 0.02] and Ile/Val [Ratio of mean Hg (95% CI) = 1.46 (1.12, 1.91), *P* = 0.01] genotypes.

**Conclusions:**

Since this is the first study from Jamaica to report these findings, replication in other populations is recommended.

**Supplementary Information:**

The online version contains supplementary material available at 10.1186/s12887-023-04452-w.

## Background

Mercury is a highly toxic omnipresent heavy metal, xenobiotic, and known neuro-toxicant [1–6]. There is no known safe limit for human Hg exposure [7]. In the environment, Hg is available in three chemical forms: elemental Hg (eHg), organic Hg (oHg), and inorganic Hg (iHg). Additionally, oHg has three forms methyl Hg (mHg), ethyl Hg (eHg), and phenyl Hg (pHg) [8, 9]. Each of these forms has a different level of toxicity and bioavailability [9]. For example, oHg is more hazardous and toxic to humans than iHg [10]. In the marine environment, iHg is transformed into highly toxic mHg by the bio-methylation process. Methyl Hg then biomagnifies up the food chain and bioaccumulates in fish [10–13]. The total blood Hg concentration (BHgC) is a valid biomarker for recent mHg exposure [14]. Hg exposure may affect the health of people of all age groups [11], however, children are at specific risk [7] as it may cause harm to their growing central nervous system (CNS) [3]. Moreover, human renal, reproductive, cardiovascular, respiratory, and immune systems may be affected by Hg [7, 11]. Adverse effects on the CNS are particularly crucial since 5–10% of infants born worldwide are affected by neurodevelopmental disorders [15, 16]. A low to moderate early childhood oral mHg exposure may cause subtle nervous system (NS) impairments, [17] and may be associated with reduced attention span, memory, language, and visual-motor skill development [18].

Heavy metals (e.g. Hg) are present in all parts of the biotic and abiotic environment [19]*.* Most important environmental Hg exposure sources are food of animal (fish) and plant (rice and vegetables) origin, pharmaceuticals and utility products (Hg fever thermometers, skin lightening lotion and creams) [3, 7, 11, 13]. Humans may be exposed to Hg through ingestion, inhalation, transdermal and trans-placental absorptions [7, 20]. Skin lightening or bleaching products use is a public health concern in many countries, including Jamaica, as these products may contain Hg [21]. Therefore, pregnant women and women of childbearing age (18–44 years old) are of special concern regarding Hg exposure through the consumption of fish as well as through the use of Hg containing skin products as they can transfer Hg to their fetuses through the placenta [21]. Although perinatal and pediatric mHg exposure are worrisome, prenatal Hg exposure is also a concern as the developing brain is very sensitive to mHg poisoning [22]. Therefore, both prenatal and early childhood Hg exposure is a critical public health concern for many countries where significantly higher amounts of fish are usually consumed.

Fish is one of the most essential and traditional dietary components for people in Jamaica because of its availability and easy accessibility [23]. According to the Food and Agricultural Organization (FAO) of the United Nations (UN) 2016 data, in Jamaica, the average per capita fish consumption was 27.1 kg/year and significantly higher than the world’s per capita supply of fish for consumption (19.7 kg/year) [24]. Ricketts et al. (2020) found the Hg levels in commonly consumed fish samples from Jamaica were between 0.09–0.32 μg/g, which were much lower than the acceptable limit of Hg level (1 μg/g) in fish set by the Food and Drug Administration (FDA) [23]. Therefore, the risk of high level Hg exposure through fish consumption was low in Jamaica [23]. Hence, there is a possibility that a significantly higher average fish consumption is responsible for the high BHgC of Jamaican children who consume specific types of fish. Additionally, geochemical investigations showed Jamaican soil was enriched with several heavy metals, including Hg (with a mean of 221 µg/kg to a maximum of 830 µg/kg), and in some areas it was higher than the world average soil Hg levels of 60 µg/kg [25–27]. Hence, there is a possibility that crops, vegetables, and fruits grown in Jamaica may contain high levels of Hg. Exposure to Hg through high per capita fish consumption and other Hg contaminated foods grown in Jamaica may pose adverse health outcomes in Jamaican children.

The glutathione S-transferase (GST) superfamily includes six genes of which *GSTT1*, *GSTM1,* and *GSTP1* are well known for their vital roles in excretory process and detoxification of xenobiotics (e.g. Hg) [28]. Several candidate genes are linked to the glutathione (GSH) detoxification system, including glutathione peroxidase and GST enzymes [4]. Glutathione has a crucial role in Hg metabolism [29]. Methyl Hg is excreted into the bile as GSH conjugates, and it is removed from the body with feces [29]. Reduced GSH production results in less excretion of Hg in the bile, which results in Hg retention [29]. GSTs are actively engaged in Hg conjugation and protect cells from oxidative stress and toxic chemicals [30, 31]. Many GST genes, including *GSTT1, GSTM1,* and *GSTP1,* are highly polymorphic [31]. *GSTT1* and *GSTM1* deletion polymorphisms have epistatic effects, which is a cause for the enhanced susceptibility to Hg exposure [4]. Moreover, null alleles of *GSTT1* and *GSTM1* result in a lack of enzyme activity [32], which causes slower Hg detoxification [33]. All this evidence suggests that genetic variations may provide an explanation for differences in BHgC in among populations.

In collaboration with faculty members at the University of the West Indies (UWI), Mona campus, in Jamaica, our research team at the University of Texas Health Science Center at Houston (UTHealth) has been examining the additive and interactive associations of six heavy metals, including Hg and GST genes (*GSTT1, GSTM1* and *GSTP1*), among children with and without autism spectrum disorder (ASD) in Jamaica since 2009. Using data from 266, 1:1 age- and sex-matched (2–8 years) ASD cases and typically developing (TD) controls, we previously investigated interactive roles of three GST genes (*GSTT1, GSTM1,* and *GSTP1*) and ASD status in relation to BHgCs of Jamaican children. In GLMs after adjusting for child’s age, socioeconomic status (SES), consumption of leafy vegetables, fried plantain, canned fish, and the interaction between *GSTP1* and *GSTT1*, we found significant interactions between *GSTP1* and children’s ASD status in relation to BHgC using either a co-dominant or dominant genetic model for *GSTP1* (*P* < 0.001, *P* = 0.007, respectively). This study found that in TD control children with the *GSTP1* Ile/Val genotype compared with ASD children the geometric mean (GM) BHgCs were significantly higher than in children with the Ile/Ile genotype (0.72 vs. 0.49 µg/L, *P* = 0.03) or the Val/Val genotype (0.72 vs. 0.51 µg/L, *P* = 0.04). These findings suggest a possible role of *GSTP1* in the detoxification of Hg that may affect the adverse health outcomes in relation to Hg exposure among the Jamaican TD children [34]. In another ERAJ study, we found a statistically significant relationship (all *P* < 0.05) between specific types of fish and seafood (i.e., saltwater fish, sardine, or mackerel fish) consumption and higher GM BHgCs in Jamaican children regardless of their ASD status; for example children who consumed seafood > 6 times per week had GM BHgC among ASD cases and controls of 1.57 µg/L and 1.31 µg/L, respectively and the GM BHgC of Jamaican children with ASD was significantly higher (0.75 μg/L) among those who ate specific types of fish than those who did not eat those fish (0.15 μg/L) [35]. However, the association between Hg exposure and its elimination and excretory mechanism influenced by the polymorphisms of GST genes and the effect of their interaction with environmental factors on BHgC are unknown. In this present study, we assessed the relationship of the most common environmental sources of Hg exposure and sociodemographic factors, as well as the possible interactions of these factors with the genotypes for the three GST genes *(GSTT1, GSTM1, GSTP1*), in relation to BHgCs in Jamaican TD children.

## Materials and methods

### General description and the study population

In this exposure assessment study, we used data from 375 TD Jamaican children (2–8 years old), who were enrolled as controls between December 2009 and September 2017, in the Epidemiological Research on Autism in Jamaica (ERAJ) and ERAJ Phase-2 (ERAJ-2), that are age-and sex-matched case control studies of ASD. Inclusion criteria for TD children in ERAJ studies included 1) the child must be born in Jamaica and 2) should be between 2–8 years old at the time of enrollment. Age and sex-matched TD controls were recruited and enrolled from schools, well-child clinics, and community churches, mostly from Kingston Parish of Jamaica [36]. The matching criteria for this study required the control children to be the same sex and to be within six months of the age of their matched ASD cases. The procedure for enrolling TD controls has been explained in detail elsewhere [37, 38]. To exclude the possibility of any developmental disorders in TD children, the life-time form of the Social Communication Questionnaire (SCQ) was administered to the parents or guardians [39, 40]. Children with SCQ scores ≤ 6 were considered for enrollment as TD controls in this study. The cut-off value of 6 was one standard deviation higher than the mean SCQ score of TD school children [41]. We also administered a SES questionnaire to the parents or guardians to collect socio-demographic and economic status information regarding children and their parents [34, 42]. A food frequency questionnaire was used to assess children’s possible Hg and other heavy metals exposure from food consumption [37]. Questions were asked about how often the child ate specific food items per week, focusing on the types and frequency of fruits, vegetables, grains, and fish and seafood consumption. For example, types of fish and seafood were classified into the following categories: saltwater fish, freshwater fish (pond fish, tilapia), canned fish (sardine or mackerel), canned tuna, salted fish (pickled mackerel), shell-fish (lobsters, crabs), and shrimp [42]. This food frequency data represented the typical current consumption of food items by children at the time of data collection [34, 35, 39]. The different types of food consumption were considered as binary categorical variables (consumed vs. never consumed) during data analysis. Blood samples (about 4–5 mL of whole blood) were collected from every child at the end of the interview and other study procedures to evaluate the exposure to heavy metals, including Hg, and to determine children’s GST genotypes.

### Assessment of Hg exposures

The BHgC analyses were conducted at the Trace Metals Lab, a Centers for Disease Control and Prevention (CDC) certified lab at the MDHHS in Lansing, Michigan, United States. The technology for metal concentrations detection in blood samples has altered over the past decade. For this reason, the MDHHS reported different limits of detection (LoD) for Hg in phases 1 and 2 of the ERAJ studies, which were 0.3 µg/L and 0.25 µg/L, respectively [34]. For quality control (QC), bovine blood spiked with known quantities of Hg was used as a control for Hg levels in the blood samples. All blood samples were diluted and analyzed on a PerkinElmer Elan DRC II inductively coupled plasma mass spectrometer (PerkinElmer, Waltham, MA, USA).

### Genetic analysis

For genetic analysis, regions of the *GSTM1* and *GSTT1* genes were amplified in two independent TaqMan Copy Number Assay reactions, *GSTM1* Assay ID: Hs02575461_cn and *GSTT1* Assay ID: Hs00010004_cn (www.thermofisher.com), to identify insertion or deletion polymorphisms. *GSTM1* and *GSTT1* homozygous deletions were coded as DD, and the presence of an insertion was coded as I* (I/I or I/D). Assessment of the *GSTP1* Ile105Val polymorphism (rs1695) was carried out using the TaqMan Drug Metabolism SNP genotyping assay C_3217198_20. All three assays have been previously described in detail [34, 39].

### Statistical analysis

Descriptive analyses were conducted to describe the distributions of demographic and SES characteristics of TD children and their parents. The BHgCs were not normally distributed in this dataset so we transformed this data using the natural logarithm (ln). Then the mean of the log transformed BHgC was changed to the original scale (μg/L) using an exponential function, herein called the GM of BHgC, that is (Exp [Mean (ln BHgC) = GM BHgC]). This study found 9.6% of BHgCs were below LoD or censored. We imputed BHgC data by dividing the LoD by the square root of two (LoD/√2) to include all the BHgCs below LoD in this data analysis [34, 43, 44].

The genotyping test for *GSTT1* and *GSTM1* does not differentiate between homozygotes (I/I) and heterozygotes (I/D); hence, only the recessive genetic model was taken into consideration for these two genes using binary variables: I* (I/I or I/D) and DD (null). For the *GSTP1* Ile105Val polymorphism, there are three genotypes: Ile/Val, Val/Val, and Ile/Ile. Analysis is possible for all three *GSTP1* genotypes. To assess the *GSTP1* genotypes, we used three genetic models, including the dominant model [Val* (Ile/Val or Val/Val) vs. Ile/Ile], co-dominant model [Ile/Ile, Ile/Val, and Val/Val], and recessive model [Val/Val vs. Ile* (Ile/Ile or Ile/Val)]. We used the Chi-square test based on available data for Jamaican TD children to determine whether the *GSTP1* polymorphism satisfies the Hardy–Weinberg equilibrium expectations. This was done due to the presence of three genotypes (Ile/Ile, Ile/Val, and Val/Val) for *GSTP1*.

We used univariable GLMs to investigate the possible additive association of various environmental exposures, three GST genes, socio-demographic characteristics, and the consumption of different types of food with log transformed BHgC as the dependent variable.

We used multivariable GLMs (both additive and interactive) to evaluate the potential gene-environment interactions between the three GST genes (*GSTT1, GSTM1,* and *GSTP1*) and various environmental factors with log transformed BHgC. In the multivariable GLMs, we only included variables that were significant in the univariable GLMs. Consequently, additive and interactive multivariable models were fitted to evaluate the relationship of the environmental factors and GST genes with log transformed BHgC, and in the final multivariable interactive GLM we only kept variables that were statistically significant. Additionally, to decrease the potential effects of multi-collinearity, we looked at pairwise correlations between all pairs of individual environmental factors that were statistically significant in the additive models. When the model became unstable by adding both correlated variables, we chose one of the variables to remain in the model. The ratios of mean Hg and 95% confidence interval (CI) for the association between the children's exposure to environmental factors and BHgC by the children's genotypes for GST genes were calculated when we identified significant gene-environment interactions using the SAS ESTIMATE statement [45]. The same method was used to calculate the ratios of the mean Hg and 95% CI for the association between the children's GST genotypes and BHgC by exposure to environmental factors. Ratios of the mean Hg were calculated as shown below using the ratios of mean Ln_Hg in children who consumed saltwater fish vs. those who did not consume saltwater fish as an example,

$$\begin{array}{c}Exp (Ln Hg) = {\beta }_{0} + {\beta }_{1} * saltwater\, fish \,consumption\\ {\beta }_{1}\hat{}= Ln\_Hg (consumed \,saltwater \,fish) - Ln\_Hg (did \,not \,consume \,saltwater \,fish)\\ = Ln [Hg (consumed \,saltwater \,fish) / Hg (did \,not \,consume \,saltwater \,fish)]\\ = EXP [Ln \{Hg (consumed \,saltwater \,fish) / Hg (did \,not \,consume \,saltwater \,fish)\}] \\ = Ratio \,of \,mean \,Hg \left(children \,consumed \,saltwater \,fish \,vs. \,those \,who \,did \,not \,consume \,saltwater \,fish\right).\end{array}$$

The significance level for all statistical tests was set at 0.05, and without corrections for multiple comparisons. All data analyses were performed using SAS 9.4 software [46].

## Results

A description of the socio-demographic features of 375 TD children and their parents, as well as children’s GST genotypes are provided in Table 1. As expected, 81.9% of TD children were male as they were matched by sex of the participating children with ASD in the ERAJ studies. About 25% of children were 72 months or older at the time of enrollment. Most children (97.3%) were Afro-Caribbean, and nearly 62% were born in Kingston parish of Jamaica. The null (DD) genotype frequency for *GSTT1* and *GSTM1* was 25.8% and 24.9% among TD children, respectively. The frequencies of the three genotypes for *GSTP1* (Ile/Ile, Ile/Val, and Val/Val) in TD children were 26.7%, 50.7%, and 22.6%, respectively, which were in agreement with Hardy–Weinberg equilibrium expectations (*P* = 0.77). At the time of the child’s birth, 11.7% of mothers were aged 35 years or more, and 44.1% of children had at least one parent with an education level beyond high school. Higher levels of SES were measured by the ownership of a car by the parents or the family of the children. At the time of enrollment 40.3% of the families had higher SES.

**Table 1:** Sociodemographic characteristics of Jamaican TD children and their parents and children’s GST genotypes (*n* = 375)

Variables		Categories	*n* (%)
**Child**	**Sex**	Male	307 (81.9)
		Female	68 (18.1)
	**Age at enrollment (months)**	Age < 72	281 (74.9)
		Age ≥ 72	94 (25.1)
	**Race**	Afro-Caribbean	365 (97.3)
	**Parish of birth**	Kingston parish	232 (61.9)
		Other parishes ^a^	143 (38.1)
	***GSTT1*** ^***b***^	DD ^c^	92 (25.8)
		I* ^d^	264 (74.2)
	***GSTM1*** ^***e***^	DD	89 (24.9)
		I*	268 (75.1)
	***GSTP1*** ^***f***^	Ile/Ile	96 (26.7)
		Ile/Val	182 (50.7)
		Val/Val	81 (22.6)
**Maternal age at child’s birth (years)** ^**g**^		Age < 35	326 (88.4)
		Age ≥ 35	43 (11.7)
**Parental education level at child’s birth** ^**h**^		Both up to high school ^i^	199 (55.9)
		At least one beyond high school ^j^	157 (44.1)
**Socioeconomic status (SES) of parents/family**		High SES (own a car)	151 (40.3)

^a^ Includes Portland, Trelawny, Westmoreland, Clarendon, St. Andrew, St. Mary, St. James, St. Elizabeth, St. Catherine, St. Thomas, St. Ann, Hanover, or Manchester parishes of Jamaica. ^b^ *GSTT1* was missing for 19 children. ^c^ DD specifies the null alleles for *GSTT1* and *GSTM1* genes. ^d^ I* (I/I or I/D) specifies the homozygote (I/I) or a heterozygote (I/D) for *GSTT1* and *GSTM1* genes. ^e^ *GSTM1* was missing for 18 children. ^f^ *GSTP1* was missing for 16 children. ^g^ Maternal age was missing for 6 mothers. ^h^ Parental education level was missing for 19 participants. ^i^ Up to high school education means attending primary/junior secondary and secondary/high/technical schools. ^j^ Beyond high school education means attending a vocational, tertiary college, or university

In the univariable GLM that assessed the association of the children’s sociodemographic characteristics, GST genotypes and exposure to various dietary and environmental factors with BHgC, we found a significant association between the children’s age and BHgC. Specifically, children aged 72 months or more had higher mean BHgC than children aged less than 72 months [Ratio of mean Hg (95% CI) = 1.25 (1.05, 1.48), *P* = 0.01]. When we assessed the association between seafood consumption and BHgCs, we found children who consumed saltwater fish and canned fish (sardine, mackerel) had higher mean BHgCs than children who did not consume those fish [Ratio of mean Hg (95% CI) = 1.40 (1.19, 1.64), *P* < 0.01; and 1.37 (1.12, 1.68), *P* < 0.01, respectively]. Similar results were found in relation to consumption of canned tuna and shrimp. Specifically, children who consumed canned tuna and shrimp had higher mean BHgCs than children who did not consume canned tuna and shrimp [Ratio of mean Hg (95% CI) = 1.21 (1.03, 1.41), *P* = 0.02; and 1.22 (1.01, 1.49), *P* < 0.05, respectively]. For beans consumption, we found children who ate peas (red peas and gungo peas) and broad beans had higher mean BHgCs compared to those who did not eat these types of food [Ratio of mean Hg (95% CI) = 1.28 (1.06, 1.54), *P* = 0.01; and 1.17 (1.01, 1.36), *P* = 0.04, respectively]. For fruits and vegetable consumption, we found significantly higher mean BHgCs in TD children who consumed leafy vegetables (lettuce) [Ratio of mean Hg (95% CI) = 1.25 (1.07, 1.45), *P* = 0.01], legumes (string beans) [Ratio of mean Hg (95% CI) = 1.31 (1.13, 1.52), *P* < 0.01], and fruit (avocado]) [Ratio of mean Hg (95% CI) = 1.21 (1.04, 1.41), *P* = 0.02] as compared to children who did not consume these fruits and vegetables. There were no significant associations between the BHgC and children’s GST (*GSTT1, GSTM1*, and *GSTP1*) genotypes (all *P* > 0.05) (see Table 2).

**Table 2:** Associations of environmental factors and children’s GST genotypes with blood Hg concentrations based on univariable General Linear Models (GLMs) (n = 375)

Exposure Variables	Category		Yes		No		Ratio of Mean Hg^**^ [Exposure Yes vs. No] (95% CI)	*P* value ^***^
			**Mean Hg** ^*^ **(µg/L)**	**N**	**Mean Hg** ^*^ **(µg/L)**	**N**		
**Child’s age (months)**	Age ≥ 72		0.88	94	0.71	281	1.25 (1.05, 1.48)	0.01
**Child’s sex**	Male		0.75	307	0.76	68	0.98 (0.81, 1.19)	0.84
**Child’s race**	Afro-Caribbean		0.75	365	0.85	10	0.87 (0.55, 1.39)	0.57
**Parish of child’s birth**	Kingston parish		0.76	232	0.73	143	1.04 (0.89, 1.21)	0.61
**Socioeconomic status (SES)**	Own a car		0.76	151	0.74	224	1.04 (0.89, 1.21)	0.66
**Maternal age at child’s birth (years)** ^**a**^	≥ 35		0.70	43	0.75	326	0.93 (0.73, 1.17)	0.53
**Parental education levels at child’s birth** ^**b**^	At least one of the parents had education beyond high school ^c^		0.71	157	0.78	199	0.91 (0.78, 1.07)	0.26
**Source of drinking water** ^**d**^	Piped water		0.74	358	0.87	16	0.85 (0.59, 1.23)	0.40
**Source of cooking water** ^**e**^	Piped water		0.74	362	0.69	11	1.07 (0.69, 1.66)	0.76
**Traffic**	Lived near a high-traffic road ^f^		0.71	145	0.77	230	0.92 (0.79, 1.08)	0.30
**Seafood consumption**	Saltwater fish		0.83	260	0.59	115	1.40 (1.19, 1.64)	< 0.01
	Freshwater fish (pond fish, tilapia)		0.83	120	0.71	255	1.16 (0.99, 1.37)	0.06
	Sardine, mackerel (canned fish)		0.79	316	0.57	59	1.37 (1.12, 1.68)	< 0.01
	Tuna (canned fish)		0.84	135	0.70	240	1.21 (1.03, 1.41)	0.02
	Salted fish (pickled mackerel		0.78	295	0.66	80	1.18 (0.98, 1.41)	0.08
	Shellfish (lobsters, crabs)		0.90	48	0.73	327	1.23 (0.99, 1.54)	0.07
	Shrimp		0.88	66	0.72	309	1.22 (1.01, 1.49)	0.045
**Organ/meat consumption**	Liver		0.79	232	0.68	143	1.16 (0.99, 1.35)	0.07
**Grain and starches consumption**	White rice or rice and peas		0.75	368	0.82	7	0.91 (0.52, 1.58)	0.74
	Fried dumpling (Festival dumpling)		0.74	304	0.79	71	0.93 (0.77, 1.13)	0.47
	Boiled dumpling		0.74	337	0.85	38	0.87 (0.68, 1.11)	0.27
	White bread		0.72	241	0.81	127	0.89 (0.76, 1.04)	0.14
	Whole wheat bread		0.77	239	0.71	136	1.09 (0.94, 1.28)	0.26
	Cakes/buns		0.74	319	0.78	56	0.95 (0.77, 1.18)	0.66
	Porridge (cornmeal, oatmeal)		0.74	343	0.84	32	0.88 (0.68, 1.15)	0.36
	Cold breakfast cereal		0.74	301	0.79	74	0.94 (0.78, 1.13)	0.50
	Pasta, macaroni, noodles		0.73	325	0.88	50	0.83 (0.67, 1.03)	0.10
**Beans consumption**	Peas, beans, Nuts	Red peas, gungo peas	0.79	299	0.62	76	1.28 (1.06,1.54)	0.01
		Broad beans	0.80	224	0.68	151	1.17 (1.01, 1.36)	0.04
		Peanuts, cashews	0.75	294	0.75	81	1.00 (0.83, 1.19)	0.97
**Fruits and vegetable consumption**	Root vegetables	Yam, sweet potato, dasheen, coco	0.75	259	0.74	116	1.02 (0.86, 1.20)	0.84
		Carrot, pumpkin	0.75	325	0.76	50	0.98 (0.78, 1.22)	0.84
	Leafy vegetables	Lettuce	0.81	234	0.65	141	1.25 (1.07, 1.45)	0.01
		Callaloo, broccoli, or pak choi	0.77	307	0.68	68	1.13 (0.93, 1.37)	0.23
		Cabbage	0.74	233	0.76	142	0.98 (0.84, 1.14)	0.81
	Legumes	String beans	0.87	162	0.67	213	1.31 (1.13, 1.52)	< 0.01
	Fruit	Tomatoes	0.76	280	0.73	95	1.04 (0.87, 1.23)	0.68
		Ackee	0.76	259	0.73	116	1.05 (0.89, 1.23)	0.58
		Avocado	0.81	229	0.67	146	1.21 (1.04, 1.41)	0.02
		Green banana	0.75	265	0.75	110	1.00 (0.85, 1.18)	0.98
		Fried plantain	0.76	318	0.70	57	1.08 (0.88, 1.33)	0.47
**Genes**	*GSTT1* (I*) ^g, i^		0.76	264	0.72	92	1.06 (0.89, 1.26)	0.51
	*GSTM1(*I*) ^g, j^		0.74	268	0.79	89	0.93 (0.78, 1.11)	0.41
	*GSTP1*(Ile/Ile) ^h, k^		0.71	96			REF	
	*GSTP1*(Ile/Val)		0.82	182	0.71	96	1.15 (0.96, 1.38)	0.13
	*GSTP1*(Val/Val)		0.66	81	0.71	96	0.92 (0.74, 1.14)	0.43

^*^ Mean Hg indicates the geometric mean of blood Hg = Exp [Mean (ln Hg)]. ^**^ Ratio of mean Hg indicates the ratio of the geometric mean blood Hg = Exp [Mean (ln Hg) in Yes vs. No exposure groups] calculated using the SAS ESTIMATE option for GLM. ^***^ *P-*values are based on GLM that compare geometric mean BHgCs between children who had the characteristic described (in the “Yes” column) vs. those who did not (in the “No” column), the “Yes” column includes participants who had the characteristic described for the categories in each variable and the “No” column includes participants who did not have those features described for the categories in each variable. ^a^ Maternal age was missing for 6 participants. ^b^ Parental education level was missing for 19 participants. ^c^ Beyond high school education means attended a vocational, tertiary college, or university. ^d^ Source of drinking water was missing for 1 participant. ^e^ Source of cooking water was missing for 2 participants. ^f^ Child lived within a quarter of a mile of a high traffic road. ^g^ I* indicates the homozygote (I/I) or a heterozygote (I/D) for *GSTT1* and *GSTM1* genes. ^h^ *GSTP1* gene has three genotypes (Ile/Ile, Ile/Val, and Val/Val). ^i^ *GSTT1* was missing for 19 children. ^j^ *GSTM1* was missing for 18 children. ^k^ *GSTP1* was missing for 16 children

Additional details regarding the descriptive analysis of distribution of BHgC before any log transformation (e.g., median and the interquartile range) are also shown as part of the supplementary materials (see Table S1).

In Table 3, we also assessed the relationship between environmental exposures and BHgC by children’s genotype for GST genes using unadjusted interactive multivariable GLMs that included the interactions between GST genes and environmental exposures in relation to BHgC. Using a co-dominant genetic model for *GSTP1*, we identified a significant interaction between the children’s birth parish and the children’s genotypes for *GSTP1* in relation to BHgCs (overall interaction *P* = 0.04). Specifically, in children with the Ile/Val genotype, TD children who were born in Kingston parish had higher mean BHgC compared to those children who were born in other parishes [Ratio of mean Hg (95% CI) = 1.28 (1.03, 1.58), *P* = 0.02]. There was no statistically significant association present between children’s birth parish and BHgC among children with Ile/Ile genotype [Ratio of mean Hg in children who were born in Kingston parish vs. those born in other parishes (95% CI) = 0.94 (0.69 -1.28), *P* = 0.69] and Val/Val genotype [Ratio of mean Hg in children who were born in Kingston parish vs. those born in other parishes (95% CI) = 0.81 (0.59, 1.12), *P* = 0.20].

**Table 3:** Associations between children’s exposure to environmental factors and BHgC by children’s genotypes for GST genes based on the multivariable General Linear Models that include the interaction between GST genes and the primary environmental exposure (n = 375)

Environmental factor		Category compared (Column B)	Referent category (Column C)	Gene	Models	Genotypes	Ratio of Mean Hg ^*^ [Column B vs Column C] (95% CI)	*P* value ^**^	Overall Interaction *P* value ^***^
**Parish of child’s birth**		Kingston	Other ^a^	*GSTP1* ^*b*^	Dominant	Val* ^c^	1.11 (0.93, 1.33)	0.25	0.37
						Ile/Ile	0.94 (0.68, 1.29)	0.70	
					Recessive	Val/Val	0.81 (0.59, 1.12)	0.20	0.07
						Ile* ^d^	1.14 (0.96 1.36)	0.15	
					Co-dominant	Ile/Ile	0.94 (0.69, 1.28)	0.69	0.04
						Ile/Val	1.28 (1.03, 1.58)	0.02	
						Val/Val	0.81 (0.59, 1.12)	0.20	
**Child lived near a high traffic road** ^**e**^		Yes	No	*GSTT1 *^*f*^	Recessive	DD ^g^	1.19 (0.87,1.63)	0.27	< 0.05
						I* ^h^	0.83 (0.69, 0.99)	0.04	
				*GSTP1*	Dominant	Val*	0.79 (0.67, 0.95)	0.01	0.01
						Ile/Ile	1.31 (0.96, 1.77)	0.08	
					Recessive	Val/Val	0.75 (0.54, 1.04)	0.09	0.22
						Ile*	0.95 (0.80, 1.13)	0.54	
					Co-dominant	Ile/Ile	1.31 (0.97, 1.76)	0.08	0.02
						Ile/Val	0.80 (0.65 0.98)	0.03	
						Val/Val	0.75 (0.54, 1.04)	0.08	
**Parental education level** ^**i**^		Group 1 ^j^	Group 2 ^k^	*GSTP1*	Dominant	Val*	0.79 (0.66, 0.95)	0.01	0.03
						Ile/Ile	1.19 (0.88, 1.60)	0.27	
					Recessive	Val/Val	0.89 (0.64, 1.24)	0.45	0.99
						Ile*	0.89 (0.74, 1.06)	0.20	
					Co-dominant	Ile/Ile	1.19 (0.88, 1.60)	0.26	0.07
						Ile/Val	0.77 (0.62, 0.96)	0.02	
						Val/Val	0.89 (0.64, 1.24)	0.50	
**Consumption**	**white bread**	Yes	No	*GSTT1*	Recessive	DD	0.63 (0.45, 0.88)	0.01	0.02
						I*	0.98 (0.81,1.18)	0.82	
	**fried dumplings**	Yes	No	*GSTM1 *^*l*^	Recessive	DD	0.62 (0.43, 0.91)	0.01	0.01
						I*	1.09 (0.87, 1.37)	0.43	
	**green banana**	Yes	No	*GSTT1*	Recessive	DD	1.37 (0.96–1.95)	0.09	0.03
						I*	0.86 (0.72, 1.04)	0.13	
	**carrot**	Yes	No	*GSTM1*	Recessive	DD	0.69 (0.47, 1.02)	0.06	0.04
						I*	1.14 (0.86, 1.50)	0.37	
	**string beans**	Yes	No	*GSTM1*	Recessive	DD	1.00 (0.74, 1.35)	1.00	0.08
						I*	1.36 (1.15, 1.63)	< 0.01	
	**canned fish (sardine, mackerel)**	Yes	No	*GSTP1 *	Dominant	Val*	1.23 (0.97, 1.57)	0.08	0.19
						Ile/Ile	1.68 (1.13 2.50)	0.01	
					Recessive	Val/Val	0.76 (0.48, 1.19)	0.22	0.01
						Ile*	1.56 (1.24, 1.95)	< 0.01	
					Co-dominant	Ile/Ile	1.68 (1.14 2.48)	0.01	0.02
						Ile/Val	1.51 (1.15 1.98)	< 0.01	
						Val/Val	0.76 (0.48, 1.19)	0.22	

^*^ Ratio of mean Hg = Exp [Mean (ln Hg)] in compared category vs. referent category of exposures calculated using the SAS ESTIMATE statement for GLM. ** *P*-values are for comparing the mean BHgC of TD children with environmental factors between the “compared category” to those with the “referent category,” stratified by the genotypes of the three GST genes, based on the SAS ESTIMATE statement for GLMs as described in the Methods section. ***Overall interaction *P*-values are based on the type 3 effect test in multivariable general linear models. ^a^ Includes Portland, Trelawny, Westmoreland, Clarendon, St. Andrew, St. Mary, St. James, St. Elizabeth, St. Catherine, St. Thomas, St. Ann, Hanover, or Manchester parishes of Jamaica. ^b^ *GSTP1* was missing for 16 children. ^c^ Val* includes Ile/Val or Val/Val genotypes of *GSTP1* gene. ^d^ Ile* includes Ile/Ile or Ile/Val genotypes of *GSTP1* gene. ^e^ Child lived within a quarter of a mile of a high traffic road. ^f^ *GSTT1* was missing for 19 children. ^g^ DD specifies the null genotypes of *GSTT1* and *GSTM1* genes. ^h^ I* (I/I or I/D) specifies the homozygote (I/I) or a heterozygote (I/D) for *GSTT1* and *GSTM1* genes. ^i^ Parental education level was missing for 19 parents. ^j^ At least one parent had beyond high school education (attended vocational, tertiary college, or university). ^k^ Both parents had up to high school education (attended primary/junior secondary or secondary/high/technical schools). ^l^ *GSTM1* was missing for 18 children

In a recessive genetic model for *GSTT1*, we identified a significant interaction between exposure to traffic and children’s genotype for *GSTT1* in relation to BHgCs (overall interaction.

*P* = 0.046). Specifically, among the TD children with I* (I/I or I/D) genotype, those who lived near a high traffic road had lower mean BHgC than those who did not live near a high traffic road [Ratio of mean Hg (95% CI) = 0.83 (0.69, 0.99), *P* = 0.04]. Whereas among children with DD (null) genotype*,* no statistically significant association was found between the exposure to traffic and BHgC [Ratio of mean Hg in children who lived near a high traffic road vs. those who did not (95% CI) = 1.19 (0.87, 1.63), *P* = 0.27].

Additionally, using a dominant genetic model for *GSTP1*, we identified a significant interaction between exposure to traffic and the children’s genotypes for *GSTP1* in relation to BHgCs (overall interaction *P* = 0.01). Specifically, TD children with Val* (Val/Val or Ile/Val) genotypes and lived near a high traffic road had lower mean BHgC than those who did not live near a high traffic road [Ratio of mean Hg (95% CI) = 0.79 (0.67, 0.95) *P* = 0.01]. On the other hand, in children with the Ile/Ile genotype, no statistically significant association was found between exposure to traffic and BHgCs [Ratio of mean Hg in children who lived near a high traffic road vs. those who did not (95% CI) = 1.31 (0.96, 1.77), *P* = 0.08]. Similarly, under a co-dominant genetic model for *GSTP1*, we identified a significant interaction between exposure to traffic and the children’s genotypes for *GSTP1* in relation to BHgC (overall interaction *P* = 0.02). Specifically, among children with Ile/Val genotype, the mean BHgC was lower for children who lived near a high traffic road than for children who did not live near a high traffic road [Ratio of mean Hg (95% CI) = 0.80 (0.65, 0.98) *P* = 0.03]. However, there was no statistically significant association between exposure to traffic and BHgCs in children with Ile/Ile [Ratio of mean Hg in children who lived near a high traffic road vs. those who did not (95% CI) = 1.31 (0.97, 1.76), *P* = 0.08] and Val/Val [Ratio of mean Hg in children who lived near a high traffic road vs. those who did not (95% CI) = 0.75 (0.54, 1.04), *P* = 0.08] genotypes.

Additionally, using a dominant *GSTP1* genetic model, we found a significant interaction between parental education level and the children’s genotype for *GSTP1* in relation to BHgC (overall interaction *P* = 0.03). Specifically, children with Val* (Ile/Val or Val/Val) genotypes who had at least one parent with education beyond high school had lower mean BHgC than children who had both parents with up to high school education [Ratio of mean Hg (95% CI) = 0.79 (0.66, 0.95), *P* = 0.01]. However, in children with Ile/Ile genotypes*,* no statistically significant association was found between parental education level and BHgC [Ratio of mean Hg in children who had at least one parent with education beyond high school vs. children who had both parents with up to high school education (95% CI) = 1.19 (0.88, 1.60), *P* = 0.27].

For ‘grain and starches consumption’, using a recessive genetic model for *GSTT1*, we found a significant interaction between consumption of white bread and the children’s genotype for *GSTT1* in relation to BHgC (overall interaction *P* = 0.02). Specifically, in TD children with DD (null) genotypes, those who ate white bread had lower mean BHgC than children who did not eat white bread [Ratio of mean Hg (95% CI) = 0.63 (0.45, 0.88), *P* = 0.01]. However, in children with I* (I/I or I/D) genotypes, there was no significant association between white bread consumption and BHgC [Ratio of mean Hg in children who ate white bread vs. those who did not (95% CI) = 0.98 (0.81, 1.18), *P* = 0.82].

Moreover, using a recessive genetic model for *GSTM1*, we identified a significant interaction between the consumption of fried dumplings and the children’s genotype for *GSTM1* in relation to BHgC (overall interaction *P* = 0.01). Specifically, in children with DD (null) genotypes, those who consumed fried dumplings had lower mean BHgC than children who did not consume fried dumplings [Ratio of mean Hg (95% CI) = 0.62 (0.43, 0.91), *P* = 0.01]. However, in children with I* (I/I or I/D) genotypes, there was no statistically significant association between fried dumpling consumption and BHgC [Ratio of mean Hg in children who consumed fried dumplings vs. those who did not (95% CI) = 1.09 (0.87, 1.37), *P* = 0.43].

For ‘fruits and vegetable’ consumption, in the recessive genetic model for *GSTT1,* we found a significant interaction between consumption of green banana and children’s *GSTT1* genotypes in relation to BHgCs (overall interaction *P* = 0.03). Moreover, using a recessive genetic model for *GSTM1*, we found a significant interaction between consumption of carrots and a marginally significant interaction between the consumption of string beans and the children’s *GSTM1* genotype in relation to BHgCs (overall interaction, *P* = 0.04 and *P* = 0.08, respectively). Specifically, among children with I* (I/I or I/D) genotypes for *GSTM1*, children who consumed string beans had higher mean BHgC compared to children who did not consume string beans [Ratio of mean Hg (95% CI) = 1.36 (1.15, 1.63), *P* < 0.01]. However, in children with the DD (null) *GSTM1* genotypes there was no significant association between string bean consumption and BHgC [Ratio of mean Hg in children who consumed string beans vs. those who did not [Ratio of mean Hg (95% CI) = 1.00 (0.74, 1.35), *P* = 1.00].

For ‘seafood consumption’ using a recessive and co-dominant genetic model for *GSTP1*, we identified significant interactions between children’s genotypes for *GSTP1* and consumption of canned fish (sardine, mackerel) in relation to BHgC (overall interaction *P* = 0.01 and *P* = 0.02, respectively). Specifically, in children with Ile* (Ile/Ile or Ile/Val) genotypes for the recessive genetic model of *GSTP1,* those who consumed canned fish (sardine, mackerel) had higher mean BHgC than those who did not consume those canned fish [Ratio of mean Hg (95% CI) = 1.56 (1.24, 1.95), *P* < 0.01]. However, in children with Val/Val genotype under the *GSTP1* recessive genetic model, there was no significant association found between the consumption of canned fish (sardines, mackerel) and BHgC [Ratio of mean Hg in children who consumed canned fish (sardines, mackerel) vs. those who did not (95% CI) = 0.76 (0.48, 1.19) *P* = 0.22]. Moreover, using the co-dominant genetic model for *GSTP1*, in children with Ile/Ile and Ile/Val genotypes, those who consumed canned fish (sardine, mackerel) had higher mean BHgC than children who did not consume these canned fish [Ratio of mean Hg (95% CI) = 1.68 (1.14, 2.48), *P* = 0.01, and 1.51 (1.15, 1.98), *P* < 0.01, respectively]. However, in children with the Val/Val genotype for the co-dominant genetic model for *GSTP1,* there was no significant association between consumption of canned fish (sardine, mackerel) and BHgC [Ratio of mean Hg in children who consumed canned fish (sardine, mackerel) vs. those who did not (95% CI) = 0.76 (0.48, 1.19), *P* = 0.22].

Additional details regarding the unadjusted association between the children’s genotype for GST genes and BHgC by environmental factors are also shown as part of the supplementary materials (see Table S2).

In Table 4, the adjusted associations between TD children’s exposure to environmental factors and their interaction with GST genes in relation to BHgC are described. In the final interactive multivariable models, we found a significant interaction between *GSTP1* genotypes and the consumption of canned fish (sardine, mackerel) in relation to BHgC using either a co-dominant or recessive genetic model (overall interaction *P* = 0.01 and *P* < 0.01, respectively). In addition, we identified the child’s age, as well as the consumption of saltwater fish, grains, and starches (pasta, macaroni, and noodles), and string beans as the other environmental factors significantly associated with BHgC (all *P* ≤ 0.04 for both adjusted models). Specifically, using the codominant genetic model for *GSTP1,* we found that children with Ile/Ile and Ile/Val genotypes who consumed canned fish (sardine, mackerel) had higher mean BHgCs compared to children who did not consume these canned fish [Ratio of mean Hg (95% CI) = 1.59 (1.09, 2.32), *P* = 0.02, and 1.46 (1.12, 1.91), *P* = 0.01, respectively].

**Table 4:** Adjusted associations between children’s exposure to environmental factors and BHgC by children’s genotypes for GST genes based on General Linear Models that include the interactions between GST genes and the main environmental exposures (n = 375)

Models	Environmental Factor		Categories compared	Gene	Genotypes	Ratio of Mean Hg* [Exposure Yes vs. No] (95% CI)	*P* value^**^
**Interactive multivariable co-dominant model**	**Child’s age (months)**		Age ≥ 72 vs. Age < 72			1.21 (1.03, 1.42)	0.02
	**Consumption**	**Saltwater fish**	Yes vs. No			1.35 (1.15, 1.59)	< 0.01
		**Pasta, macaroni, noodles**	Yes vs. No			0.72 (0.58, 0.89)	< 0.01
		**String beans**	Yes vs. No			1.17 (1.01, 1.36)	0.04
		**Canned fish (sardine, mackerel)** ^**a**^	Yes vs. No	*GSTP1*^*b*^	Ile/Ile	1.59 (1.09, 2.32)	0.02
					Ile/Val	1.46 (1.12, 1.91)	0.01
					Val/Val	0.72 (0.47, 1.11)	0.14
**Interactive multivariable recessive** **model**	**Child’s age (months)**		Age ≥ 72 vs. Age < 72			1.21 (1.03, 1.43)	0.02
	**Consumption**	**Saltwater fish**	Yes vs. No			1.35 (1.15, 1.58)	< 0.01
		**Pasta, macaroni, noodles**	Yes vs. No			0.72 (0.58, 0.89)	< 0.01
		**String beans**	Yes vs. No			1.18 (1.01,1.37)	0.03
		**Canned fish (sardine, mackerel) **^**c**^	Yes vs. No	*GSTP1*^*b*^	Val/Val	0.72 (0.46, 1.12)	0.14
					Ile*	1.50 (1.20, 1.87)	< 0.01

^*^Ratio of mean Hg indicates geometric mean BHgC ratio = Exp [Mean (ln Hg) in Yes vs. No exposure groups calculated using SAS ESTIMATE statement for GLM. **P-values are for the comparison of geometric mean BHgC of children with environmental factors in comparison of exposed (Yes) vs. non-exposed (No). ^a^ Overall interaction *P* = 0.01. ^b^ *GSTP1* was missing for 16 children. ^c^ Overall interaction *P* < 0.01. ^d^ Ile* includes Ile/Val or Ile/Ile genotypes of *GSTP1* gene

However, in children with the Val/Val genotype under the co-dominant genetic model for *GSTP1*, there was no significant association between the consumption of canned fish (sardine, mackerel) and BHgC [Ratio of mean Hg in children who consumed canned fish (sardine, mackerel) vs. those who did not (95% CI) = 0.72 (0.47, 1.11), *P* = 0.14]. In the co-dominant genetic model, we also found that in children aged 72 months or older, the mean BHgC was higher than in children aged less than 72 months [Ratio of mean Hg (95% CI) = 1.21 (1.03, 1.42), *P* = 0.02]. Moreover, TD children who consumed saltwater fish and string beans had higher mean BHgCs compared to those who did not consume those foods [Ratio of mean Hg (95% CI) = 1.35 (1.15, 1.59), *P* < 0.01, and 1.17 (0.58, 0.89), *P* = 0.04, respectively]. On the other hand, children who consumed grain and starches (pasta, macaroni, and noodles) had lower mean BHgC than children who did not consume those foods, [Ratio of mean Hg (95% CI) = 0.72 (0.58, 0.89), *P* < 0.01].

We also identified similar findings using the recessive genetic model for *GSTP1*. We found that children with Ile* (Ile/Ile or Ile/Val) genotypes who consumed canned fish (sardine, mackerel) had higher mean BHgC than children who did not consume those canned fish [Ratio of mean Hg (95% CI) = 1.50 (1.20, 1.87), *P* < 0.01]. However, in children with the Val/Val genotype under the recessive genetic model for *GSTP1,* there was no statistically significant association between consumption of canned fish (sardine and mackerel) and BHgCs [Ratio of mean Hg in children who consumed canned fish (sardine, mackerel) vs. those who did not (95% CI) = 0.72 (0.46, 1.12), *P* = 0.14].

More details about the adjusted associations between the children’s GST genotypes and BHgC are shown as part of the supplementary materials (see Table S3).

## Discussion

The findings from this study suggest that the consumption of saltwater fish and string beans has resulted in a 35% and 17% higher mean BHgC among Jamaican children who consumed those foods compared to those who did not consume those foods, respectively. On the other hand, the consumption of grain and starches (pasta, macaroni, and noodles) was associated with a 28% lower mean BHgC in Jamaican children who consumed those foods compared to those who did not consume those foods. In addition, we found that children aged 72 months and older had a 21% higher mean BHgC than the group of younger children. The association between the consumption of canned fish (sardine, mackerel) and BHgCs varied by children’s *GSTP1* genotypes, using either a recessive or a co-dominant genetic model (overall interaction.

*P* < 0.01 and *P* = 0.01, respectively). The consumption of canned fish (sardine, mackerel) was significantly associated with 59% and 46% higher mean BHgCs only among children with *GSTP1* Ile/Ile, and Ile/Val genotypes respectively. We discuss the main study findings separately in the following sections.

### Role of child’s age in BHgC

When comparing our findings with the GM BHgC of 7–11-year-old children in other countries, we observed that the GM BHgC of these TD Jamaican children is near the level reported in children in some European countries, but compared with Ecuador and China is 3–4 times lower (see Table S4).

Our study also showed that children’s age is significantly associated with GM BHgC among Jamaican TD children, which is consistent with a study conducted in the United States that showed the GM BHgC among children aged 1–5 years was 0.34 µg/L [95% CI, (0.30, 0.39) µg/L] and in women aged 16–49 years was 1.02 µg/L [95% CI, (0.85, 1.20) µg/L] [14]. Another study in New Zealand provided similar evidence of increased BHgC with increasing age. This study showed that the GM BHgCs among children and adults were 0.86 µg/L and 1.65 µg/L respectively [47].

All of this evidence supports the present study findings of 1.21 times higher mean BHgC among TD children aged 72 months and older compared to children in the younger age group (*P* = 0.02). The reason behind this phenomenon may be the slow clearance of Hg from the human body, which causes the BHgC to gradually increase as children get older [48]. Confirmation and dissemination of this study finding that mean BHgC may gradually increase with child’s age is warranted for the parents in countries where large amounts of fish and seafood are consumed like Jamaica in order to help protect their children from neurodevelopmental disorders by reducing and limiting their children’s Hg exposure through more suitable food choices.

### Association of seafood consumption and BHgCs

Our study finding of 1.35 times higher mean BHgC in children who consumed saltwater fish compared with children who did not consume saltwater fish is consistent with a study conducted in New Zealand that demonstrated fish consumption three or more times per week was associated with 2.7 times higher total BHgC in children when compared to eating fish less than once per week [47]. Moreover, another study in China showed children who consumed saltwater fish 4 to 6 times per week had a significantly higher odds ratio for BHgCs compared to children who ate saltwater fish less than or equal to 1–3 times per month in both adjusted and unadjusted models (*P* < 0.01) [49]. A previous study in Jamaica demonstrated the median of BHgC for TD children (266 children) when adjusted for the frequency of seafood consumption, maternal age, and parental education was 0.55 [39].

Fish and seafood are good sources of low-fat protein [50], and rich in various necessary vitamins and minerals [50]. For example, selenium found in fish has a crucial role in fetal brain development, growth, thyroid hormone metabolism, calcium regulation, and prevention or reversal of oxidative damage [23, 51]. Fish and seafood are also vital sources of nutrients such as essential fatty acids, including omega-3 and omega-6 fatty acids [50], eicosapentaenoic acid (EPA) and docosahexaenoic acid (DHA) [52, 53]. These essential fatty acids have been associated with reduced risk of cardiovascular disease, heart attack [23], and coronary deaths [52]. Additionally, these essential fatty acids are beneficial for early neurodevelopment [52, 54], and also have been implicated in preventing some specific types of cancers [55]. However, in addition to the enormous health benefits of fish and seafood consumption, there are also risks of exposure to chemical contaminants such as heavy metals [23]. Fish is the primary source of Hg exposure, especially mHg for many populations [35, 53, 56–58]. For example, a study conducted in Taiwan showed that saltwater fish was the primary dietary exposure for mHg among women of childbearing age [53]. A report titled “Seafood Choices: Balancing Risks and Benefits” by the US Institute of Medicine recommends that although fish and seafood can replace other protein sources with greater levels of saturated fat, it is still important for consumers to choose food carefully that will reduce or limit their exposure to pollutants like mHg that are present in fish and seafood [59].

It was mentioned previously that fish is the traditional and most essential dietary component for people of the Caribbean island nation, Jamaica, where the average per capita fish consumption (27.1 kg/year) [60] was higher than the world’s average per capita food fish supply (19.7 kg/year) [24]. As a result, information about exposure data for toxic heavy metals (e.g., Hg) through the higher average amount of fish consumption would be helpful for the Jamaican public health authorities to generate to increase awareness about Hg exposure in their country. Based on the previous study findings in Jamaica, the policies of Jamaica's Ministry of Health and Wellness and Ministry of Education and Youth, in consultation with the Planning Institute of Jamaica, were altered, and children are no longer served canned fish (sardine or mackerel) in Jamaican public schools [34].

These present study findings will contribute additional knowledge on fish and seafood consumption and their effects on BHgC of Jamaican children who are one of the most vulnerable populations for developing adverse health outcomes, specifically neurodevelopmental disorders from Hg exposure.

### Consumption of fruit and vegetables and BHgCs

Mercury concentrations are high in the surface soil in Jamaica compared to other countries, with a mean of 221 µg/kg to a maximum of 830 µg/kg [25, 27, 61]. For example, the guideline limits for agricultural land use with Hg levels in the soil of Canada and Denmark are 6.6 µg/kg and 3 µg/kg, respectively [25]. Moreover, there is a possibility of Hg contamination of surface water in Jamaica due to anthropogenic activities and other industrial activities such as bauxite processing [61]. Previous studies provided evidence that plant roots absorb Hg from the soil and plant leaves absorb Hg from the air [62]. Therefore, consuming vegetables grown in Jamaican soil polluted with organic Hg (mainly mHg) may cause significant risks to human health. Haixin Yu et al. (2018) have reported in their meta-analysis that beans, such as string beans or long beans, absorb Hg from the soil, although the bio-concentration factor (BCF) is lower than for other vegetables such as green peppers and spinach [62]. These findings are consistent with this present study findings that children who consumed string beans had 1.17 times higher mean BHgC than children who did not consume string beans (*P* = 0.04). This finding suggests the possibility that consumption of string beans and other crops, fruits and vegetables grown in highly Hg-contaminated Jamaican soil may pose an increased risk of higher mean BHgC in Jamaican children. So, further study of Hg concentrations of different crops grown in Jamaica and their possible effects on BHgC of Jamaican children is warranted to make parents aware about proper food choices for their children to lower their Hg exposure through food.

### Role of GST genes in BHgCs of Jamaican children with and without consumption of canned fish (sardine, mackerel)

One of the distinctive features of this present study is the availability of genetic data on Jamaican children. Data are available for children’s genotypes for the *GSTT1, GSTM1,* and *GSTP1* genes. Detailed description of the distribution of these three GST genotypes for Jamaica and a few other countries are provided as part of the supplementary materials (see Table S5). While investigating the possible interactive effects of *GSTP1* genotypes and the consumption of canned fish (sardine, mackerel) in relation to BHgCs in Jamaican children after controlling for environmental factors such as consumption of saltwater fish, string beans, and consumption of grain and starches (pasta, macaroni, and noodles), as well as the children’s age we found that the *GSTP1* gene could be an effect modifier for the association between the consumption of canned fish (sardine, mackerel) and BHgCs when using either a co-dominant or recessive genetic model.

Though the detoxification efficiency or capacity has not been examined or tested in this study, the association we found between the *GSTP*1 Ile105Val polymorphism and a lower BHgC suggests a better Hg detoxification capacity among children having two Val alleles (Val/Val) for the *GSTP1* Ile105Val polymorphism who consumed canned fish (sardine, mackerel). This finding is consistent with the finding of the previous study by Rahbar et al., (2021) in Jamaica. That study showed that under the co-dominant genetic model for *GSTP1*, TD children with Val/Val genotypes had a lower GM BHgC than children with Ile/Val genotypes (0.51 µg/L vs. 0.72 µg/L *P* = 0.04) [34].

Currently, there is a knowledge gap in available information on the effect of genetic predisposition on metal toxicokinetics (i.e. how the body metabolizes and eliminates a toxic metal) in the human body [63, 64]. However, the evidence that suggests specific genetic variants affect Hg toxicokinetics is growing [63, 64]. For example, cytochrome P450 monooxygenases (CYP450) catalyze the oxidation and metabolism of a large number of xenobiotics including Hg. CYP450 enzymes evolved as the primary defense against xenobiotics (Hg) and during this process are also responsible for the bioactivation of toxicants to a more reactive intermediate [65]. Furthermore, GSH plays a crucial role in Hg metabolism [29]. Experiments have shown that mHg exposure could cause depleted concentrations of antioxidant molecules such as tripeptide glutathione (GSH) and reduced activities of important antioxidant enzymes, such as catalase (CAT), glutathione-peroxidase (GPx), and superoxide dismutase (SOD) that might lead to oxidative damage in lipids, proteins, and DNA [64]. Some genes are related to the GSH detoxification systems, namely genes coding for enzymes involved in glutathione metabolism as well as GPx and GST enzymes. The intracellular binding reaction with GSH is catalyzed by the various GST isoenzymes and leads to the formation of stable GSH-metal (e.g., Hg) conjugates, which are transported out of the cells and excreted from the human body via feces and urine [66, 67]. Increased Hg retention in blood and plasma, as well as increased urinary excretion, may be linked to *GSTP1* polymorphisms with reduced or no conjugation capability [29]. A study conducted in Sweden showed that study participants who carried at least one Val allele for the *GSTP1* Ile105Val polymorphism had lower Hg concentrations in erythrocytes [68]. To the best of our knowledge, we are the first to report an interactive association between *GSTP1* Ile105Val and the consumption of canned fish (sardine, mackerel) in relation to BHgC among Jamaican, 2–8 years old TD children. Therefore, the validation of this finding in other populations is warranted.

### Limitations

We acknowledge several limitations to this study. The first limitation is that this study’s participants were the TD control children from the ERAJ studies selected to match the ASD cases by age and sex and were more likely to be enrolled from the Kingston parish of Jamaica. As a result, around 82% and 62% of the study participants (TD children) were male and born in Kingston parish of Jamaica, respectively. However, one of the most consistent findings from previous studies of ASD is the 4:1 male to female ratio [69]. Therefore, participants in this study may not represent a random sample from the general population of all children in Jamaica. As a result, the findings regarding BHgCs described here may not be generalizable to all Jamaican children.

Secondly, the BHgCs data from the ERAJ studies did not distinguish between oHg and iHg exposures. Therefore, this study does not provide a complete overview of different sources of Hg exposure.

Moreover, although maternal exposure to Hg during pregnancy through fish consumption and using Hg containing skin bleaching and whitening products are also important sources of Hg exposure for their children, we did not have any data regarding these kinds of exposures.

In addition, since data on the quantity of food items consumed was not available in this study, we used a binary (consumed vs. never consumed) approach for food exposures.

Furthermore, fish consumption is a crucial source of Hg exposure in Jamaica because of the presence of significant amounts of fish in the traditional Jamaican diet. Although we used a standard and culturally appropriate food frequency questionnaire to measure the frequency of fish and seafood consumption, the questionnaire did not separate the frequency of different types of fish and seafood consumption. This limits the assessment of individual associations of different kinds of fish or seafood consumption with BHgC in Jamaican TD children. For example, we have information on how many sardines or mackerel fish servings children consumed per week, but this study did not assess the frequency of sardine and mackerel consumption individually. This is crucial because, according to Silbernagel et al. (2011), mackerel is one of the highest mHg contaminated fish while sardines are considered to have lower mHg concentrations [70].

In univariable models, we found that the consumption of peas (red peas, gungo peas), broad beans, leafy vegetables (lettuce), and fruit (avocado) were positively associated with BHgCs. However, due to the presence of associated independent variables in the regression models, these dietary items were eliminated from the multivariable analysis to reduce the possibility of multicollinearity. Fruits and vegetables are significant sources of nutritional factors, including vitamins, minerals, and fibers. However, many of these fruits and vegetables are also contaminated with heavy metals such as Hg, arsenic, and others. Moreover, fruits and vegetables should make up one-half of the plate at each meal according to the 2015–2020 US Dietary Guidelines for Americans [71]. We would encourage more research on exploring the Hg levels in different food items and the risk–benefit assessment of each type of fruit and vegetable consumed in Jamaica.

Although several studies have shown rice is one of the most crucial sources of Hg exposure in many regions, we did not find any association between rice consumption and BHgC among Jamaican TD children, possibly because nearly all of our study participants (98%) consumed rice. It is possible that some of the participants ate imported food from other countries. However, this option was not included in the food frequency questionnaire used for this study, which means that the notion that most of the food items consumed by the study participants were also locally cultivated and caught remains in doubt. Furthermore, without examining the health outcomes that are associated with these exposures, the results from this study should be interpreted with great caution especially when being used for food safety considerations and advisories. Data's delay owing to the date of sample collection is another weakness for this study. Moreover, considering the half-life of Hg, using a single sample for BHgC evaluation is another limitation of this study.

Moreover, based on n = 375, our calculations indicate that we have 83% power to detect small effect-sizes of 0.3 or higher at 5% level of significance. However, since this study is a secondary exploratory analysis, we didn’t adjust for multiple comparisons. Accordingly, we recommend that further investigation of these reported relationships be conducted in other country and population settings with larger sample sizes, adjusted for multiple comparisons, and using data for Hg levels in commonly consumed food categories and fish in Jamaica.

## Conclusion

In this study, we identified the child’s age, as well as consumption of saltwater fish, canned fish (sardine, mackerel), string beans, grain, and starches (pasta, macaroni, noodles), as the environmental factors significantly associated with BHgCs in Jamaican TD children.

Based on the adjusted multivariable analysis, which included gene-environment interaction, the findings suggest that only children with Ile/Ile and Ile/Val genotypes for the *GSTP1* Ile105Val polymorphism who consumed canned fish (sardine, mackerel) had a higher mean BHgC compared to children who did not consume those canned fish. This finding implies that having two Val alleles (Val/Val) for the *GSTP1* Ile105Val polymorphism is associated with more effective detoxification of Hg in Jamaican children who ate canned fish (sardine, mackerel). Therefore, increasing the understanding among parents regarding how these dietary and environmental factors could affect BHgCs will potentially lead to lower Hg exposure among Jamaican children, especially among those who are more susceptible to adverse health outcomes related to Hg exposures due to their genetic predispositions.

### Supplementary Information

**Supplementary file 1:** **Table S1.** Distribution of Blood Hg Concentration (Median, IQR) with Different Environmental Exposures and GST Genotypes among Jamaican Children. **Table S2.** Associations between children’s GST genotypes and BHgC by exposure to environmental factors based on the multivariable General Linear Models that include the interactions between GST genes and the main environmental exposures (n = 375). **Table S3.** Association between children’s genotype for *GSTP1* and BHgC by consumption of canned fish (sardine, mackerel) based on General Linear Models that adjust for child’s age, consumption of saltwater fish, grain and starches (pasta, macaroni, and noodles), and string beans. **Table S4.** Geometric Mean (GM) Blood Mercury Concentration (BHgC) among 7-11 years old children in Different Countries*. **Table S5.** GST Gene (*GSTT1, GSTM1, GSTP1*) Genotypes Distribution in Different countries.

## Data Availability

The data analyzed in this study are from two grants (i.e., R21 and R01). The data from R01 are or will be publicly available through the National Database for Autism Research (NDAR). Data from R21 will also be available upon request from the corresponding author.

## Abbreviations

Hg: Mercury
BHgC: Blood Hg concentration
GST: Glutathione S-transferase
TD: Typically developing
eHg: Elemental Hg
oHg: Organic Hg
iHg: Inorganic Hg
mHg: Methyl Hg
eHg: Ethyl Hg
pHg: Phenyl Hg
NS: Nervous system
FAO: Food and Agricultural Organization
FDA: Food and Drug Administration
GM: Geometric mean
GSH: Glutathione
ASD: Autism spectrum disorder
UWI: University of the West Indies
UTHealth: The University of Texas Health Science Center at Houston
ERAJ: Epidemiological Research on Autism in Jamaica
SCQ: Social Communication Questionnaire
SES: Socioeconomic status
CDC: Centers for Disease Control and Prevention
LoD: Limits of Detection
QC: Quality control
GLMs: General linear models
CI: Confidence interval
EPA: Eicosapentaenoic acid
DHA: Docosahexaenoic acid
CAT: Catalase
GPx: Glutathione-peroxidase
SOD: Superoxide dismutase
