# Supplementary material for: Factors associated with blood mercury concentrations and their interactions with three glutathione S-transferase genes (GSTT1, GSTM1, and GSTP1): an exposure assessment study of typically developing Jamaican children
Source: BMC Pediatr. 2024 Jan 4;24:14. doi: 10.1186/s12887-023-04452-w (PMC10765857; doi:10.1186/s12887-023-04452-w)
Supplement: Supplementary file 1 — Table S1. Distribution of Blood Hg Concentration (Median, IQR) with Different Environmental Exposures and GST Genotypes among Jamaican Children. Table S2. Associations between children’s GST genotypes and BHgC by exposure to environmental factors based on the multivariable General Linear Models that include the interactions between GST genes and the main environmental exposures (n = 375). Table S3. Association between children’s genotype for GSTP1 and BHgC by consumption of canned fish (sardine, mackerel) based on General Linear Models that adjust for child’s age, consumption of saltwater fish, grain and starches (pasta, macaroni, and noodles), and string beans. Table S4. Geometric Mean (GM) Blood Mercury Concentration (BHgC) among 7-11 years old children in Different Countries*. Table S5. GST Gene (GSTT1, GSTM1, GSTP1) Genotypes Distribution in Different countries. [file 12887_2023_4452_MOESM1_ESM.docx]

**Factors Associated with Blood Mercury Concentrations and Their Interactions with Three Glutathione S-Transferase Genes (*GSTT1*, *GSTM1*, and *GSTP1*): An Exposure Assessment Study of Typically Developing Jamaican Children**

Sheikh Farzana Zaman^1,2^, Maureen Samms-Vaughan^3^, Sepideh Saroukhani^2,4^, Jan Bressler^1,5^, Manouchehr Hessabi^2^, Megan L. Grove^1,5^, Sydonnie Shakespeare Pellington^3^, Katherine A. Loveland^6^, Mohammad H. Rahbar^1,2,4,*^

^1^ Department of Epidemiology, Human Genetics, and Environmental Sciences (EHGES), School of Public Health, The University of Texas Health Science Center at Houston, Houston, TX 77030, USA

^2^ Biostatistics/Epidemiology/Research Design (BERD) Component, Center for Clinical and Translational Sciences (CCTS), The University of Texas Health Science Center at Houston, Houston, TX 77030, USA

^3^ Department of Child & Adolescent Health, The University of the West Indies (UWI), Mona Campus, Kingston 7, Jamaica

^4^ Division of Clinical and Translational Sciences, Department of Internal Medicine, McGovern Medical School, The University of Texas Health Science Center at Houston, Houston, TX 77030, USA

^5^ Human Genetics Center, School of Public Health, The University of Texas Health Science Center at Houston, Houston, TX 77030, USA

^6^ Louis A Faillace, MD, Department of Psychiatry and Behavioral Sciences, McGovern Medical School, The University of Texas Health Science Center at Houston, Houston, TX 77054, USA

* Correspondence: mohammad.h.rahbar@uth.tmc.edu; Tel.: +1-713-500-7901; Fax: +1-713-500-076

* **Correspondence:** [mohammad.h.rahbar@uth.tmc.edu](mailto:mohammad.h.rahbar@uth.tmc.edu);

Tel.: +1-713-500-7901; Fax: +1-713-500-0766

| **Table S1. Distribution of Blood Hg Concentration (Median, IQR) with Different Environmental Exposures and GST Genotypes among Jamaican Children** | | | | | | | |
| --- | --- | --- | --- | --- | --- | --- | --- |
| **Exposure Variables** | **Category** | | | **Median Hg (µg/L)** | **Q1*** | **Q3**** | **N** |
| **Child’s age (months)** | Age ≥ 72 | | | 0.92 | 0.62 | 1.50 | 94 |
|  | Age <72 | | | 0.75 | 0.48 | 1.10 | 281 |
| **Child’s sex** | Male | | | 0.78 | 0.49 | 1.20 | 307 |
|  | Female | | | 0.75 | 0.51 | 1.15 | 68 |
| **Child’s race** | Afro-Caribbean | | | 0.78 | 0.49 | 1.20 | 365 |
|  | Others | | | 0.80 | 0.59 | 0.99 | 10 |
| **Parish of child’s birth** | Kingston parish | | | 0.80 | 0.49 | 1.20 | 232 |
|  | Other parishes | | | 0.76 | 0.49 | 1.10 | 143 |
| **Socioeconomic status (SES)** | Own a car | | | 0.82 | 0.48 | 1.30 | 151 |
|  | Did not own a car | | | 0.77 | 0.50 | 1.10 | 224 |
| **Maternal age at child’s birth (years) ^a^** | ≥35 | | | 0.65 | 0.44 | 1.10 | 43 |
|  | <35 | | | 0.78 | 0.49 | 1.20 | 326 |
| **Parental education levels at child’s birth ^b^** | At least one of the parents had education beyond high school ^c^ | | | 0.76 | 0.47 | 1.20 | 157 |
|  | Both up to high school | | | 0.80 | 0.49 | 1.20 | 199 |
| **Source of drinking water ^d^** | Piped water | | | 0.79 | 0.49 | 1.10 | 358 |
|  | Other than piped water | | | 0.63 | 0.52 | 1.04 | 16 |
| **Source of cooking water ^e^** | Piped water | | | 0.79 | 0.49 | 1.10 | 362 |
|  | Other than piped water | | | 0.61 | 0.48 | 0.77 | 11 |
| **Traffic** | Lived near a high-traffic road ^f^ | | | 0.70 | 0.47 | 1.10 | 145 |
|  | Did not live near a high-traffic road | | | 0.81 | 0.52 | 1.20 | 230 |
| **Food consumption** | | | | | | | |
| **Fish and Seafood consumption** | Saltwater fish | | Yes | 0.83 | 0.54 | 1.30 | 260 |
|  |  |  | No | 0.59 | 0.34 | 0.99 | 115 |
|  | Sardine, mackerel (canned fish) | | Yes | 0.81 | 0.54 | 1.20 | 316 |
|  |  |  | No | 0.50 | 0.32 | 1.10 | 59 |
|  | Tuna (canned fish) | | Yes | 0.88 | 0.56 | 1.20 | 135 |
|  |  |  | No | 0.75 | 0.44 | 1.10 | 240 |
|  | Shrimp | | Yes | 0.84 | 0.59 | 1.30 | 66 |
|  |  |  | No | 0.76 | 0.48 | 1.10 | 309 |
| **Grain and starches consumption** | White rice or rice and peas | | Yes | 0.78 | 0.49 | 1.15 | 368 |
|  |  |  | No | 0.64 | 0.54 | 1.60 | 7 |
|  | White bread | | Yes | 0.78 | 0.48 | 1.10 | 241 |
|  |  |  | No | 0.82 | 0.52 | 1.40 | 127 |
|  | Pasta, macaroni, noodles | | Yes | 0.76 | 0.49 | 1.10 | 325 |
|  |  |  | No | 0.90 | 0.54 | 1.60 | 50 |
| **Beans consumption** | Peas, beans,  Nuts | Red peas, gungo peas | Yes | 0.81 | 0.52 | 1.20 | 299 |
|  |  |  | No | 0.64 | 0.34 | 1.10 | 76 |
|  |  | Broad beans | Yes | 0.83 | 0.54 | 1.25 | 224 |
|  |  |  | No | 0.71 | 0.44 | 1.10 | 151 |
| **Fruits and vegetable consumption** | Leafy vegetables | Lettuce | Yes | 0.83 | 0.54 | 1.30 | 234 |
|  |  |  | No | 0.68 | 0.37 | 1.10 | 141 |
|  | Legumes | String beans | Yes | 0.84 | 0.55 | 1.30 | 162 |
|  |  |  | No | 0.71 | 0.39 | 1.10 | 213 |
|  | Fruit | Avocado | Yes | 0.81 | 0.54 | 1.30 | 229 |
|  |  |  | No | 0.75 | 0.37 | 1.10 | 146 |
| **GST Genes** | | | | | | | |
| **Genes** | *GSTT1* | | (I*) ^g, i^ | 0.79 | 0.51 | 1.25 | 264 |
|  |  |  | *DD ^g, i^* | 0.79 | 0.49 | 1.10 | 92 |
|  | *GSTM1* | | (I*) ^g, j^ | 0.77 | 0.49 | 1.10 | 268 |
|  |  |  | *DD ^g, j^* | 0.81 | 0.54 | 1.30 | 89 |
|  | *GSTP1*(Ile/Ile) ^h, k^ | | Yes | 0.76 | 0.45 | 1.10 | 96 |
|  |  |  | No | 0.81 | 0.52 | 1.10 | 263 |
|  | *GSTP1*(Ile/Val) ^h, k^ | | Yes | 0.84 | 0.52 | 1.20 | 182 |
|  |  |  | *No* | 0.75 | 0.48 | 1.10 | 177 |
|  | GSTP1(Val/Val) ^h, k^ | | Yes | 0.72 | 0.51 | 0.99 | 81 |
|  |  |  | No | 0.81 | 0.49 | 1.30 | 278 |
| * Lower quartile (25th percentile) ** Upper quartile (75th percentile) ^a^ Maternal age was missing for 6 participants. ^b^ Parental education level was missing for 19 participants. ^c^ Beyond high school education means attending a vocational, tertiary college, or university. ^d^ Source of drinking water was missing for 1 participant. ^e^ Source of cooking water was missing for 2 participants. ^f^ Child lived within a quarter of a mile of a high traffic road. ^g^ I* indicates the homozygote (I/I) or a heterozygote (I/D) for *GSTT1* and *GSTM1* genes. ^h^ *GSTP1* gene has three genotypes (Ile/Ile, Ile/Val, and Val/Val). ^i^ *GSTT1* was missing for 19 children. ^j^ *GSTM1* was missing for 18 children. k *GSTP1* was missing for 16 children. | | | | | | | |

| **Table S2. Associations between children’s GST genotypes and BHgC by exposure to environmental factors based on the multivariable General Linear Models that include the interactions between GST genes and the main environmental exposures (n = 375)** | | | | | | | | |
| --- | --- | --- | --- | --- | --- | --- | --- | --- |
| **Gene** | **Model** | **Genotypes**  **Compared (Column C)** | **Referent genotypes (Column D)** | **Environmental factor** | **Category** | **Ratio of Mean Hg^*^ [Column C vs. Column D]  (95% CI)** | ***P* value^**^** | **Overall Interaction *P* value^***^** |
| ***GSTT1*^a^** | Recessive | DD ^b^ | I* ^c^ | Child lived near a high traffic road ^c^ | Yes | 1.19 (0.89, 1.58) | 0.25 | 0.05 |
|  |  |  |  |  | No | 0.82 (0.66, 1.02) | 0.07 |  |
|  | Recessive | DD ^b^ | I* ^c^ | Consumption of white bread | Yes | 0.83 (0.67, 1.02) | 0.08 | 0.02 |
|  |  |  |  |  | No | 1.28 (0.93, 1.76) | 0.12 |  |
|  | Recessive | DD ^b^ | I* ^c^ | Consumption of green banana | Yes | 1.06 (0.87- 1.30) | 0.57 | 0.03 |
|  |  |  |  |  | No | 0.67 (0.47, 0.95) | 0.03 |  |
| ***GSTM1*^d^** | Recessive | DD ^b^ | I* ^c^ | Consumption of fried dumpling | Yes | 0.96 (0.79, 1.17) | 0.70 | 0.01 |
|  |  |  |  |  | No | 1.69 (1.14 2.50) | 0.01 |  |
|  | Recessive | DD ^b^ | I* ^c^ | Consumption of carrot | Yes | 0.99 (0.82, 1.20) | 0.91 | 0.04 |
|  |  |  |  |  | No | 1.63 (1.05, 2.51) | 0.03 |  |
|  | Recessive | DD ^b^ | I* ^c^ | Consumption of string bean | Yes | 0.90 (0.69 -1.17) | 0.44 | 0.08 |
|  |  |  |  |  | No | 1.23 (0.98, 1.55) | 0.08 |  |
| ***GSTP1*^e^** | Dominant | Val*^f^ | Ile/Ile | Child lived near a high traffic road ^h^ | Yes | 0.78 (0.59, 1.04) | 0.09 | 0.01 |
|  |  |  |  |  | No | 1.29 (1.05, 1.59) | 0.02 |  |
|  | Recessive | Val/Val | Ile*^g^ |  | Yes | 0.72 (0.54, 0.97) | 0.03 | 0.22 |
|  |  |  |  |  | No | 0.91 (0.73, 1.14) | 0.40 |  |
|  | Co-dominant | Ile/Val | Ile/Ile |  | Yes | 0.85 (0.63, 1.13) | 0.26 | 0.02 |
|  |  | Val/Val | Ile/Ile |  |  | 0.64 (0.45, 0.92) | 0.01 |  |
|  |  | Ile/Val | Val/Val |  |  | 1.32 (0.97, 1.79) | 0.07 |  |
|  |  | Ile/Val | Ile/Ile |  | No | 1.39 (1.11, 1.73) | <0.01 |  |
|  |  | Val/Val | Ile/Ile |  |  | 1.12 (0.86, 1.45) | 0.42 |  |
|  |  | Ile/Val | Val/Val |  |  | 1.24 (0.98, 1.58) | 0.07 |  |
|  | Dominant | Val*^f^ | Ile/Ile | Parish of child’s birth | Kingston | 1.14 (0.93, 1.41) | 0.21 | 0.37 |
|  |  |  |  |  | Other ^i^ | 0.97 (0.72, 1.30) | 0.82 |  |
|  | Recessive | Val/Val | Ile*^g^ |  | Kingston | 0.73 (0.58, 0.92) | 0.01 | 0.07 |
|  |  |  |  |  | Other ^i^ | 1.02 (0.77, 1.35) | 0.87 |  |
|  | Co-dominant | Ile/Val | Ile/Ile |  | Kingston | 1.30 (1.04, 1.62) | 0.02 | 0.04 |
|  |  | Val/Val | Ile/Ile |  |  | 0.86 (0.66, 1.12) | 0.26 |  |
|  |  | Ile/Val | Val/Val |  |  | 1.52 (1.18, 1.94) | <0.01 |  |
|  |  | Ile/Val | Ile/Ile |  | Other ^f^ | 0.96 (0.70, 1.30) | 0.77 |  |
|  |  | Val/Val | Ile/Ile |  |  | 0.99 (0.69, 1.41 | 0.96 |  |
|  |  | Ile/Val | Val/Val |  |  | 0.96 (0.72, 1.29) | 0.81 |  |
|  | Dominant | Val*^f^ | Ile/Ile | Parental education level (At least one of the parents had education beyond high school) | Group 1 ^j^ | 0.88 (0.68 -1.14) | 0.32 | 0.03 |
|  |  |  |  |  | Group 2 ^k^ | 1.31 (1.03 -1.67) | 0.03 |  |
|  | Recessive | Val/Val | Ile* ^g^ |  | Group 1 ^j^ | 0.84 (0.64, 1.10) | 0.21 | 0.99 |
|  |  |  |  |  | Group 2 ^k^ | 0.84 (0.65, 1.09) | 0.19 |  |
|  | Co-dominant | Ile/Val | Ile/Ile |  | Group 1 ^j^ | 0.92 (0.70, 1.21) | 0.55 | 0.07 |
|  |  | Val/Val | Ile/Ile |  |  | 0.80 (0.58, 1.10) | 0.17 |  |
|  |  | Ile/Val | Val/Val |  |  | 1.15 (0.87, 1.53) | 0.33 |  |
|  |  | Ile/Val | Ile/Ile |  | Group 2 ^k^ | 1.42 (1.11 1.82) | 0.01 |  |
|  |  | Val/Val | Ile/Ile |  |  | 1.06 (0.78, 1.45) | 0.70 |  |
|  |  | Ile/Val | Val/Val |  |  | 1.34 (1.02, 1.76) | 0.04 |  |
|  | Dominant | Val*^f^ | Ile/Ile | Consumption of canned fish (sardine, mackerel) | Yes | 1.02 (0.85, 1.23) | 0.80 | 0.19 |
|  |  |  |  |  | No | 1.39 (0.91, 2.13) | 0.13 |  |
|  | Recessive | Val/Val | Ile* ^g^ |  | Yes | 0.75 (0.62, 0.91) | <0.01 | 0.01 |
|  |  |  |  |  | No | 1.54 (0.97, 2.46) | 0.07 |  |
|  | Co-dominant | Ile/Val | Ile/Ile |  | Yes | 1.14 (0.94, 1.38) | 0.19 | 0.02 |
|  |  | Val/Val | Ile/Ile |  |  | 0.82 (0.65, 1.02) | 0.08 |  |
|  |  | Ile/Val | Val/Val |  |  | 1.40 (1.14, 1.71) | <0.01 |  |
|  |  | Ile/Val | Ile/Ile |  | No | 1.27(0.82, 1.97) | 0.29 |  |
|  |  | Val/Val | Ile/Ile |  |  | 1.81 (1.04, 3.14) | 0.03 |  |
|  |  | Ile/Val | Val/Val |  |  | 0.70 (0.43, 1.14) | 0.15 |  |
| * Ratio of mean Hg indicates the ratio of the geometric mean blood Hg = Exp [Mean (ln Hg) with genotypes in compared category vs. referent category calculated using the SAS ESTIMATE statement for GLM. ** *P*-values are for the comparison of the mean BHgC of children with genotypes in compared category to those with the “referent category” stratified by the presence or absence of environmental factors, based on the ESTIMATE option in the SAS program for GLMs as described in the Methods section^.^ ^***^ Overall interaction *P*-values based on the type 3 effect test in multivariable generalized linear models. ^a^ GSTT1 was missing for 19 children. ^b^ DD means the null genotypes of *GSTT1* and *GSTM1*. ^c^ I* means I/I or I/D indicate the homozygote (I/I) or a heterozygote (I/D) for *GSTT1* and *GSTM1*. ^d^ GSTM1 was missing for 18 children. ^e^ GSTP1 was missing for 16 children ^f^ Val* includes Ile/Val or Val/Val genotypes of *GSTP1*. ^g^ Ile* includes Ile/Ile or Ile/Val genotypes of *GSTP1*. ^h^ Child lived within a quarter of a mile of a high traffic road. ^i^ Includes Portland, Trelawny, Westmoreland, Clarendon, St. Andrew, St. Mary, St. James, St. Elizabeth, St. Catherine, St. Thomas, St. Ann, Hanover, or Manchester parishes of Jamaica. ^j^ At least one beyond high school (beyond high school education means attended a vocational, tertiary college, or university). ^k^ Both up to high school (up to high school education means attended primary/junior secondary, and secondary/high/technical schools). | | | | | | | | |

| **Table S3. Association between children’s genotype for *GSTP1* and BHgC by consumption of canned fish (sardine, mackerel) based on General Linear Models that adjust for child’s age, consumption of saltwater fish, grain and starches (pasta, macaroni, and noodles), and string beans.** | | | | | | |
| --- | --- | --- | --- | --- | --- | --- |
| **Models** | **Environmental Factor** | **Category** | **Gene** | **Genotypes** | **Ratio of Mean Hg* [Column E] (95% CI)** | ***P* value^**^** |
| Co-dominant | Consumption of canned fish (sardine, mackerel) ^a^ | Yes | *GSTP1^b^* | Ile/Val vs. Ile/Ile | 1.14 (0.95, 1.37) | 0.15 |
|  |  |  |  | Val/Val vs. Ile/Ile | 0.78 (0.63, 0.97) | 0.03 |
|  |  |  |  | Ile/Val vs. Val/Val | 1.46 (1.21,1.77) | <0.01 |
|  |  | No |  | Ile/Val vs. Ile/Ile | 1.24 (0.82, 1.89) | 0.30 |
|  |  |  |  | Val/Val vs. Ile/Ile | 1.73 (1.02, 2.94) | 0.04 |
|  |  |  |  | Ile/Val vs. Val/Val | 0.72 (0.45, 1.15) | 0.17 |
| Recessive | Consumption of canned fish (sardine, mackerel) ^c^ | Yes | *GSTP1^b^* | Val/Val vs. Ile* ^d^ | 1.39 (1.16, 1.67) | <0.01 |
|  |  | No |  | Val/Val vs. Ile* ^d^ | 0.67 (0.43, 1.05) | 0.08 |
| * Ratio of mean Hg indicates the geometric mean BHgC ratio = Exp [Mean (ln Hg) in test genotype vs. referent genotypes calculated using the SAS ESTIMATE option in GLM. ***P*-values are for the comparison of the geometric mean BHgC of children with test genotype vs. referent genotypes. ^a^ Overall interaction *P-*value = 0.01. ^b^ *GSTP1* was missing for 16 children. ^c^ Overall interaction *P*-value <0.01. ^d^ Ile* includes Ile/Val or Ile/Ile genotypes of *GSTP1* gene. | | | | | | |

| **Table S4. Geometric Mean (GM) Blood Mercury Concentration (BHgC) among 7-11 years old children in Different Countries*** | | |
| --- | --- | --- |
| **City, Country** | **N** | **GM BHgC ** (µg/L)** |
| Koprivnica, Croatia | 52 | 0.44 |
| Prague, Czech Republic | 21 | 0.21 |
| Wroclaw, Poland | 30 | 0.12 |
| Ban. Bystrica, Slovakia | 57 | 0.52 |
| Ljubljana, Slovenia | 45 | 0.94 |
| Landskrona, Sweden | 41 | 0.43 |
| Guiyang, China | 29 | 2.45 |
| Camilo Ponce Enríquez, Ecuador | 69 | 3.23 |
| Fez, Sefrou, Morocco | 39 | 0.31 |
| * Hrubá et al., 2012 [1] **GM BHgC = Geometric mean blood Hg concentrations. | | |

| **Table S5. GST Gene (*GSTT1, GSTM1, GSTP1*) Genotypes Distribution in Different countries** | | | | | | | |
| --- | --- | --- | --- | --- | --- | --- | --- |
| **Country** | **GST genotypes (%)** | | | | | | |
|  | ***GSTT1*** | | ***GSTM1*** | | ***GSTP1*** | | |
|  | I* ^a^ | DD ^b^ | I* ^a^ | DD ^b^ | Ile/Ile | Ile/Val | val/Val |
| **Jamaica** (Presented study) | 74.2 | 25.8 | 75.1 | 24.9 | 26.7 | 50.7 | 22.6 |
| **Brazil** (Rossini et al., 2002 ) [2] | 74.6 | 25.4 | 57.9 | 42.1 | 49.7 | 38.1 | 12.2 |
| **Argentina** (Weich et al., 2017) [3] | 83.0 | 17.0 | 55.0 | 45.0 | - | - | 11.0 |
| **Ecuador** (Custodio et al., 2005) [4] | 70.0 | 30.0 | 57.0 | 43.0 | 22.0 | 50.0 | 28.0 |
| **Tunisia** (Gara et al., 2010) [5] | 83.4 | 16.6 | 65.4 | 34.6 | - | - | - |
| ^a^ I* (I/I or I/D) specifies the homozygote (I/I) or a heterozygote (I/D) for *GSTT1* and *GSTM1* genes. ^b^ DD specifies the null alleles for *GSTT1* and *GSTM1* genes. | | | | | | | |

**References**

1. Hrubá F, Strömberg U, Černá M, Chen C, Harari F, Harari R, et al. Bloodcadmium, mercury, and lead in children: an international comparison of cities in six European countries, and China, Ecuador, and Morocco. Environ Int. 2012;41:29–34.
2. Rossini A, Rapozo DCM, Amorim LMF, Macedo JMB, Medina R, Neto JFN, et al. Frequencies of GSTM1, GSTT1, and GSTP1 polymorphisms in a Brazilian population. Genet Mol Res. 2002;1:233–40.
3. Weich N, Roisman A, Cerliani B, Aráoz HV, Chertkoff L, Richard SM, et al. Gene polymorphism profiles of drug-metabolising enzymes GSTM1, GSTT1 and GSTP1 in an Argentinian population. Ann Hum Biol. 2017;44:379–83.
4. Custodio HM, Harari R, Gerhardsson L, Skerfving S, Broberg K. Genetic influences on the retention of inorganic mercury. Arch Environ Occup Health. 2005;60:17–23.
5. Gara S, Abessi M, Bendjemena K, Abdennebi M, Guemira F. Deletion polymorphism of glutathione S-transferases M1 and T1 in the Tunisian population. Tunis Med. 2010;88:700–2.
